# Current data and strategy in glioblastoma multiforme

**Published:** 2009-11-25

**Authors:** G Iacob, EB Dinca

**Affiliations:** * ‘ Carol Davila’ University of Medicine and Pharmacy, Bucharest Romania; ** Neurosurgery Clinic – Universitary Hospital BucharestRomania

**Keywords:** glioblastoma multiforme (GBM), surgical strategy, chemotherapy, experimental approaches, recurrency treatment

## Abstract

Glioblastoma multiforme (GBM) or astrocytoma grade Ⅳ on WHO classification is the most aggressive and the most frequent of all primary brain tumors. Glioblastoma is **multiforme **, resistant to therapeutic interventions illustrating the heterogeneity exhibited by this tumor in its every aspect, including clinical presentation, pathology, genetic signature.

Current data and treatment strategy in GBM are presented focusing on basic science data and key clinical aspects like surgery, including personal experience; adjuvant modalities: radiotherapy, chemotherapy, but also for experimental approaches. Therapeutic attitude in recurrent GBM is also widely discussed.

## Introduction

Malignant brain tumors are among the cancers with the most dismal prognosis and are additionally one of the most expensive cancers to treat [1]. The main culprit for adult CNS neoplasm is metastasis from other sites (especially lung, GI tract, breast cancer). Distinct from these, one encounters several types of primary brain malignancies, with tumors arising from glial cells (gliomas) being the most common. Glioblastoma multiforme (GBM) or grade 4 astrocytoma by the classification of World Health Organization is the most aggressive and frequent of all primary brain tumors [2]; the term ‘multiforme’ illustrate the heterogeneity exhibited by this tumor in its every aspect, including clinical presentation, pathology, genetic signature and response to different therapies [3].

## Epidemiology

GBM accounts for 12–15% of all brain tumors and 50–60% of astrocytomas. The incidence of GBM is less than 10 to 100,000, but the median survival of a little over 1 year from diagnosis makes it a considerable public health issue [3]. Interestingly, the incidence is fairly constant worldwide, leading to the logical inference that environmental, geographical and nutritional factors probably don't play a major role in this particular cancer, where genetics is more likely to tip the scale of etiology. The peak incidence is between 45 and 70 years, with a crest to 58 years; only 8.8% of children with central nervous system tumors had GBM [4] and congenital cases are extremely rare. A troubling observation, GBM incidence is increasing in all age groups, especially in adults, which cannot be explained by the aging population, better imaging techniques or earlier detection; no familial predisposition was found [3]. It is slightly more common in men, with a male–to female ratio of 1.5:1 – the reasons for this gender distribution are yet unknown [3]. Blacks are somewhat protected, as their incidence is lower than other ethnic groups like whites, latinos and Asians [3]. A study has found GBM patients are generally born in winter, particularly in the month of February (40%); a perinatal viral origin (infection with an unknown oncovirus, integrating in the genome) has subsequently been put forward, but no hard evidence has yet been found to support it. [3]. The association with an oncogenic virus remains controversial; anecdotically, SV40 or cytomegalovirus have been involved [3]. Most GBM appear to be sporadic, although several genetic disorders are associated with increased incidence, including tuberous sclerosis, neurofibromatosis type 1 and type 2, von Hippel Lindau disease, Turcot and Li–Fraumeni syndromes [3]. There is a proven association between GBM and exposure to ionizing radiation or polyvinyl chloride (a polymer commonly used in construction), but no links have been found between GBM and smoking, diet, cellular phones or electromagnetic fields [5].

## Historical annotations

1863 – Virchow, cited by [3] was the first one to recognize the glial origin of this tumor. 1888–Bramwell [6] is pointing out the surgical dilemma created by the highly infiltrative nature of GBM: ‘the tumor tissue is never limited by a capsule and it is impossible to say without microscopical examination where the tumor tissue ceases and the normal brain tissue begins’. 1914– Mallory [7] coined the term ‘glioblastoma multiforme’. 1926–Bailey and Cushing definitively changed the name from spongioblastoma multiforme to GBM, term also preferred by Zulch, Russel and Rubinstein [3]. 1940 –Scherer and Kernohan recognized GBM sometimes develops by progression from a lower grade lesion; the idea was later on sustained by molecular studies showing a characteristic sequential accumulation of genetic alterations from diffuse astrocytoma (WHO grade Ⅱ) to anaplastic (grade Ⅲ) and to GBM. Most cases, however, are thought to arise de novo. Scherer [8] has also paid due attention to the migration of tumor cells away from the main tumor mass through the brain parenchyma, describing the ‘secondary structures of Scherer’.

## Clinical considerations

Occasionally, GBM is asymptomatic until it reaches an enormous size. History is generally short, less than a few months and not uncommonly symptoms begin abruptly; in more than 50% of cases, this is explained by the rapid development of increased intracranial pressure, with compression and infiltration of the surrounding brain [3]. Physical findings depend on the location, size and rate of growth of the tumor, as with any other CNS tumor: headache, partial or generalized seizures in 30–60% of patients, contralateral slowly progressive hemiparesis, sudden onset of hemiplegia with depressed level of consciousness–as a stroke in evolution [9], generated by intratumoral bleeding, directly correlated with the level of expression of vascular endothelial growth factor (VEGF) [10];  sensory disturbances,  subtle personality changes, visual field defects. As survival gets longer, an increasing number of patients experience: cognitive problems, neurologic deficits resulting from radiation necrosis, communicating hydrocephalus, occasionally cranial neuropathies, polyradiculopathies from leptomeningeal spread.

## Genetics

Overexpression of several oncogenes and mutations leading to loss of function of key tumor suppressor genes concur to create one of the most aggressive cancers. Amplification of oncogenes such as the epidermal growth factor receptor (EGFR), as well as loss of tumor suppressors like PTEN on chromosome 10, p53 on hromosome 17, or p16/INK4A are some of the most common genetic alterations in GBM [11, 12]. Adult malignant astrocytoma was one of the first cancers shown to have frequent TP53 mutations [13, 14]. The p53 disfunction disrupts the downstream p14ARF pathway, impedes the process of apoptosis and fosters further genomic instability. Most commonly, the mutations occur with loss of the remaining wild–type allele, such that the tumor cells express only aberrant p53 [14]. This distribution of mutations is highly similar to those indicated in other human cancers and in which codons 175, 248, 273 are revealed as the most common targets of alteration, contributing substantially to the overall high incidence of alterations in p53's DNA binding domain. GBMs are often classified with respect to their clinical history: either as primary GBM, without indication of association with a lower malignancy precursor or as secondary GBM, having evolved from a lower grade astrocytoma. For the latter, which constitute <10% of all GBM, TP53 mutations are frequent and occur in nearly two–thirds of the cases [14]. For the more common primary GBM, only 25–30% of the cases have p53 mutations. In total and without subclassification, approximately two thirds of all GBM have wild–type p53 status. The clinical division in primary and secondary GBM is also relevant from a basic science point of view, as different genetic events are incriminated in their development [3]. For primary GBM, the main factors are considered to be EGFR amplification, loss of PTEN and INK4A [16]; all occurring almost simultaneously or in a brief period of genomic instability, correlating with a rapid clinical history, often shorter than 3 months. For secondary GBM, there is a distinct succession of genetic events and the course is longer: initially, activation of signal transduction pathways, usually through overexpression of PDGF, PDGFR, FGF2 (instead of EGFR); p53 mutation, CDK4 amplification, Rb (retinoblastoma gene) loss and eventually, loss of PTEN unleashes the Akt pathway, leading to downstream activation of mTOR (mammalian target of rapamycin) and other genes (NF–kB, an important transcription factor promoting proliferation; GSK3; anti–apoptotic genes such as BAD etc.) with devastating consequences [16]. The main way of activating Ras in GBM is through EGFR (either overexpressed and stimulated by exogenous growth factors, or constitutively active [17] as is the case for certain mutant variants, like the truncated EGFRⅢ) but binding of different growth factors to other tyrosine kinase receptors is another important upstream event. For the rare pediatric GBM, it is believed that EGFR signaling is revived up by overexpression of YB–1 (Y–Box binding protein 1). Things, unfortunately, are incredibly more complex than the deregulation of a few signaling pathways. For instance, differential recruitment of mRNAs to polysomes (thus optimizing protein synthesis) contributes to glioma formation, being an important player in the oncogenic Ras and Akt signaling [18]. Such observations lead to cell–existential questions like [3]: transcriptional control versus translational control, which is more essential to cell transformation? Does aberrant regulation of translation lead to cancer? Would it be a cause or consequence of cancer progression?

## Pathology

GBM is preferentially located supratentorially–fewer than 50 cerebellar cases have been described in literature [3], in the cerebral hemispheres, more often in the frontal lobes than the temporal lobes or basal ganglia (although a combined fronto–temporal mass is particularly typical of GBM). GBM of the brainstem (malignant brain stem glioma) are quite infrequent and often affect children [19], while spinal cord is a rare site for this neoplasm–if it occurs, the cervical and thoracic segments are most likely to be affected; when concomitant with a brain mass, some possibly develop as a ‘drop pseudo–metastasis’ from tumor cells that invaded the ventricular system. It usually presents as an irregular mass in the white matter, infiltrating the surrounding parenchyma by coursing along white matter tracts. Although tumor cells are considered to be already disseminated at time of diagnosis far in the surrounding parenchyma, it is generally a single mass, while true multifocal glioblastoma usually have distinct histological appearance and are most likely policlonal, usually presenting as simultaneous infra and supratentorial masses–incidence of 2,5% [20, 21]. Most GBMs are intraparenchymal with an epicenter in the white matter, some are largely superficial, in contact with the leptomeninges and dura, but without invading the subarachnoid space. Tipically unilateral, involving much of an entire lobe, not uncommonly it involves the corpus callosum and crosses the midline to produce the characteristic ‘butterfly’ appearance of bilateral invasion. Grossly, it appears topographically diffuse, a poorly delineated mass with no capsula, with prominent areas of old and recent hemorrhage (extensive areas of yellowish–brown to red discoloration) and necrosis (as much as 80% of the total tumor mass), cystic areas sometimes alternating with firm tissue. Microscopically, characteristic histopathological features include: cellular and nuclear polymorphism, nuclear atypia, high mitotic activity, vascular trombosis, microvascular proliferation and necrosis, with regions of pseudopalisading (created by viable tumor cells bordering areas of necrosis). Among the above, the presence of necrosis within the tumor is considered crucial, showing the aggressiveness of the cells (they outgrow the blood supply, regardless of their angiogenic potential) and it is a sine–qua–non diagnostic criterion to histopathologically ‘upgrade’ an anaplastic astrocytoma to GBM. Another important diagnostic clue is the presence of secondary structures such as perineuronal and perivascular ‘satellitosis‘ –a result from the interaction of glioma cells with host brain structures, especially in certain regions, like the subpial zone of the cortex, in the subependymal region, and through the white matter tracts (intrafascicular spread, considered a ‘favorite’ route of migration and invasion for the tumor cells). The cell composition is heterogenous (again – ‘multiforme’), especially for GBM resulting from progression of diffuse astrocytomas (grade Ⅲ): fusiform, round, pleomorphic (small, undiferentiated, lipidized, granular, giant) astrocytes–may reflect the emergence of a new tumor phenotype through stepwise acquisition of genetic alterations. Histologic tumor variants which do not alter the prognosis of the tumor (with the exception of gliomatosis who has no surgical management and a more accelerated course), include: giant cell glioblastoma, gliosarcoma and gliomatosis cerebri. Extension within and along perivascular spaces are common, but invasion of the vessel lumen seems to occur infrequently, correlating with a very low incidence of haematogenous spread to extraneural tissues. Metastatic spread of GBM occurs in less than 5% of cases, late in the illness course and it was almost unheard of before the adjuvant therapy. Dissemination within the CSF pathways is so rare that it does not seem to justify the use of prophylactic irradiation of the spinal cord [3]. In practice, GBM metastasis is considered an exceptional event. Local dissemination into subcutaneous tissues of the scalp, face and neck can occur if dura is not closed at the time of surgery. Penetration of dura is possible, although not frequent, while invasion of venous sinuses and bone is exceptional; peritoneal metastasis via ventriculoperitoneal shunt is also a possibility [3].

## Imaging

MRI with and without contrast is the most sensitive and specific study, particularly useful in evaluating tumor extension and subacute and chronic hemorrhage collections [3]. On T_1_–weighted images, the mass has low–signal intensity, presenting as a central hypodensity surrounded by a thick enhancing rim of tumor with thick, irregular walls, corresponding to the cellular and highly vascularized peripheral area of the neoplasm. Marked gadolinium enhancement indicates angiogenesis and vascular permeability. The tendency to infiltrate along the white matter tracts and involvement or crossing of the corpus callosum can also be observed. On T_2_–weighted images, it appears as a high–signal intensity mass, however the area is broader, less well defined and overlaps with the surrounding vasogenic edema; this imaging modality is best at revealing the perilesional edema. The CT scan appearance is variable and less informative and cannot replace the MRI as study of choice despite advantages of cost and time [3]. It typically shows irregularly shaped lesions with peripheral ring–like zone of contrast enhancement around a dark, central area of necrosis, usually inhomogeneous. Surrounding edema has a hypodense or isodense appearance. Functional imaging, such as positron– emission tomography (PET scan), single–photon emission computed tomography or MR spectroscopy, although cost– prohibitive for routine clinical practice, may provide useful information. The level of regional consumption of radioactively labeled glucose (used in PET scanning) correlates with cellular metabolism and reduced survival. These techniques may help differentiate the tumor from other benign mass lesions, brain abscess, toxoplasmosis and help refine the differential diagnosis between treatment-related radiation necrosis versus tumor recurrence, so difficult to distinguish in MRI or otherwise (e.g. levels of choline and lipids are different on MR spectroscopy). They may also be helpful to define the margins of the tumor for surgical resection, planning for the radiation fields and to improve the diagnostic accuracy in case a small biopsy sample is taken [3].

## Differential diagnosis

Other *primary brain tumors (meningioma, metastasis, astrocytoma, oligodendroglioma)*; features making GBM a more likely diagnosis include the irregular shape of the mass, the central necrosis, the extensive surrounding edema and mass effect. *Metastasis* should always be considered; it is less likely when the patient is realtively young and has no history of a primary tumor. *Brain abscess* should also be mentioned but it is typically identifiable as a thin, regular rim of enhancement around a central cavity. Other infections (toxoplasmosis, cysticercosis) may also have a close radiologic appearance. *Primary CNS* lymphoma may be, occasionally, ‘butterfly–shaped’ involving the corpus callosum. *Multiple sclerosis lesions* – ‘concentric sclerosis of Balo’, a borderline rare form of multiple sclerosis [22] may be difficult to separate from GBM by clinical presentation and radiologic appearance.

## Treatment

Several factors concur to make GBM treatment notoriously difficult. First, the tumor cells themselves, despite their relatively rapid cycle, are quite resistant to conventional therapies. In addition, brain has a limited capacity to repair itself, any damage may be definitive and consequential. Last but not least, before the advent of temozolomide (TMZ), adequate penetration of the blood–brain barrier (BBB) by chemotherapeutics could not be achieved without dose–limiting systemic side effects [3]. The mainstay of therapy consists of surgery, radiation and chemotherapy. Objectives of surgery range from merely confirming the diagnosis (biopsy), to alleviating symptoms of mass effect and ICP (debulking or cytoreductive surgery, resecting as much as it is safe without worsening patient's neurologic deficits), to aggressive attempts to improve not only the quality of life, but also influence survival significantly. In addition to tumor–targeted therapy, one has to treat several associated phenomena [3].

***Peritumoral edema*** may respond to a potent corticosteroid (Dexamethasone) given 4 to 10 mg every 4 to 6 h, diminishing mass effect and lowering intracranial pressure, with a decrease in headache and drowsiness.

***Seizures treatment*** is required to only 40% of patients. An appropriate anticonvulsant, with minimal side effects and cytochrome P450 interference (enzyme inducers can increase the metabolism and clearance of some chemotherapeutic agents), should first be tried as monotherapy.

***Prevention of thromboembolic disease*** is a major concern for patients with GBM, as the incidence has been reported to be as high as 35–40%. Prophylactic use of anticoagulation has not been recommended because of increased risk of intracranial hemorrhage; alternatives include appropriate mobilization and physical therapy, calf protection such as SCDs (sequential compressive devices) and radio– interventional placement of an inferior vena cava filter (Greenfield filters).

***Occupational, speech therapy, emotional and psychological support*** are also important, especially as the emphasis shifts to palliative and supportive care (a point reached, unfortunately, in the evolution of a majority of GBM patients).

## Surgery

Bennett and Godlee are credited with the first successful removal of a glial tumor (1884), cited by Iacob [3]. The extent of surgical resection depends on location and eloquence of the brain areas involved, but surgery is always an incomplete debulking, since GBM is a highly infiltrating tumor and cannot be resected completely. In a seminal study by Wilson [23], the percentage of tumor cells in the entire cell population was quantified as a function of distance from the ‘visible’ tumor edge and the averages were found to be 6% at 0–2 cm away (hence, the margin considered for ‘radical’ resection should not be less than 2 cm) and more troubling, 1.8% for 2–4 cm and 0.2% at more than 4 cm away (e.g., in the contralateral hemisphere). Whether aggressive, ‘radical’ surgery prolongs survival is still debatable, but several studies suggest a close inverse correlation between survival and the amount of residual tumor observed on postoperative MRI scans [24]. Partisans of radical resection maintain several advantages, such as: good relief of ICP, reversal of some neurologic deficits, lowering seizure incidence or even abolishing them, a definitive pathology diagnosis by reducing sampling error and the assumption that a ‘more cytoreductive’ surgery may facilitate adjuvant treatment modalities and ultimately improve survival. Arguments against radical resection stem from the inherent invasiveness of GBM, which cannot be totally resected anyway; in addition, there might be a potential for facilitating tumor cells migration by the act of surgery and the possibility of surgical complications, new neurological deficits (thinking to ‘primum non nocere’, ‘first, do no harm’). If pursued, radical resection may be improved by careful pre-operative planning, use of intraoperative MRI or at least 3D – image guidance for tumor delineation and electrophysiological mapping to help preserve eloquent areas; also, use of 5–aminolevulinic acid (ALA) for influencing fluoresceine–guided resections has recently been reported to increase survival (median of 17.7 months vs. 12.9 months) [25]. The routine use of robot arms has not yet been proven to influence survival [3].

## Radiation therapy

Early clinical trials revealed a modest, yet undeniable efficacy of external beam radiotherapy (RT) in treating GBM, based on the damage of ionizing radiation in the DNA helix by electrons and free radicals. RT is usually started within 4 to 6 weeks after surgery [26] and administered in a standard fractionated regimen over 6 to 7 weeks [27, 28].  The standard dose of external beam RT is 60 Gy in single daily fractions of 1.7–2 Gy, 5 times a week, applied to a limited field that includes the enhancing volume on CT scans with a 2–3 cm margin or 2 cm margin beyond Flair T_2_–weighted MR images. Whole brain RT does not improve survival when compared to the more precise and targeted three–dimensional conformal RT. Targeted variants of RT have been developed that are so focused, that they are closer to surgery – hence their generic name, radiosurgery. The Gamma knife has been introduced half a century ago by Lars Leksell and is now at its 4th generation, the Leksell Perfexion (introduced in clinical practice in 2007), boasting 201 sources of 60Co. These devices triangulate hundreds of gamma rays (of low individual energy) in a single spot, so ‘ground zero’ receives a very high dose of radiation, with a sharp decline in exposure for the surrounding tissue. The Cyberknife and LINAC are other highly focused radiosurgery systems. This technique is generally used to treat small (<3 cm), radiographically well–defined lesions in surgically inaccessible or eloquent areas of the brain or in patients with serious coexisting medical illnesses, unsuitable for open resection. Growth of the tumor during the course of radiation is an extremely poor prognostic sign. A major reason for radioresistance is the significantly lower oxygenation of tumors compared to surrounding cortex [29]. This may be quantified by measuring the level of a transcription factor, hypoxia–inducible factor–1 alpha (HIF–1a), a hypoxic sensor that is also an ominous indicator of tumor radioresistance. Stereotactic brachytherapy involves using stereotactic techniques to accurately place radioactive isotopes: 125I, 252Californium within the tumor. Typically, brachytherapy delivers an additional 50–60 Gy of radiation, bringing the total dose of radiation up to 110–120 Gy. This is indicated in patients with unifocal, well– defined, supratentorial tumors less than 5 cm in diameter that do not involve the corpus callosum, brain stem or ependymal surfaces; currently as a salvage modality, in recurrent GBM after repeat resection of the tumor. Recently, instead of solid seeds, a variant has been developed where a temporary balloon catheter filled with liquid radiation is implanted – the GliaSite RTS (radiotherapy system). The device is an inflatable, silicone, balloon catheter that is inserted in the resection cavity and filled with an aqueous solution of organically–bound 125I (Iotrex), which delivers 40–60 Gy at 0.5–1 cm from the balloon surface over a course of 3–7 days. Another adjuvant modality, thermotherapy, involves microwave antennas operating at 2450 or 915 MHz to deliver heat for one hour at 450C before and after 3–5 days of interstitial brachytherapy. Boron neutron capture therapy (BNCT) is an experimental form of RT where the damage occurs through the interaction between a beam of thermally slowed neutrons (created in a mini–nuclear reactor) with boron–10, which is injected to a patient and preferentially binds to tumor cells. Still investigational and costly, this is a treatment modality with great promise: Hatanaka [30] reported a 5% year survival rate of 50% with few complications, after combining intra–arterial polyhedral boron anion with focused thermal neutron radiation. The mean survival after optimal surgery and adjuvant RT (60 Gy) is about a year: 12.1 months in one of the latest studies [27]. There are several limitations to RT that make adjuvant chemotherapy a must: the infiltrative nature of GBM, the risk of radiation necrosis and radiation–induced permanent neuronal damage (e.g. radiation encephalopathy), as well as the radio–resistance of some tumors, intrinsic or acquired.

## Chemotherapy

In attempts to further improve survival beyond that offered by RT, many chemotherapeutics have been tested for effectiveness in the treatment of GBM. Among these, alkylating agents have demonstrated some benefit; either chloroethylating drugs like carmustine (BCNU), lomustine (CCNU) or methylating agents like temozolomide (TMZ), are used in the majority of GBM clinical protocols [31]. The chloroethylating agents readily penetrate the blood-brain barrier due to high hydrophobicity and they can also be administered orally [32]. They act by forming O^6^–chloroethylguanine lesions, which lead to G–C interstrand crosslinks [33], after a mean time of only 10 hours (8–12h) [34]. It is thought that as few as 2 to 5 such lesions (interstrand crosslinks) can trigger apoptosis in a tumor, as well as a normal, cell [35, 36]. However, an aggressive regimen with these agents causes considerable side effects [37], leading to dose reductions and corresponding decreases in therapeutic efficacy. Methylating agents such as TMZ show reduced toxicity toward normal cells and are much better tolerated. Oral administration of TMZ, either concomitant with radiotherapy, followed by adjuvant TMZ or as adjuvant TMZ alone after completion of radiation, is increasingly becoming standard of care for GBM patients, at least in those countries that can afford the high cost of TMZ therapy [37, 38]. The use of TMZ has been significantly increased as a result of a phase Ⅲ trial that showed survival advantage to newly diagnosed GBM patients receiving TMZ with standard radiotherapy [27]. Regarding its mechanism of action, O^6^–methylguanine (O^6^–mG) is perhaps the most biologically relevant lesion generated by TMZ; consequently, TMZ therapy has little effect in tumors where the added methyl group is removed due to the intact enzymatic activity of O^6^–methylguanine–DNA–methyltransferase (MGMT) [31, 39– 41]. The importance of MGMT epigenetic silencing through promoter methylation was shown as a favorable prognostic factor for improved response to TMZ in a multi–institutional study of GBM patients [28]. However, additional analysis of the results of this study [28] showed that 10% of the patients having tumors with non–methylated MGMT survived more than 2 years, which is considered long–term survival for GBM patients. This observation indicates that MGMT methylation status is not the sole predictor of GBM response to TMZ and this drug of choice should not be withheld from any GBM patient, unless enrolled in investigational trials. Second–line cytotoxic agents, for patients who do not respond to the first–line drugs discussed above, include carboplatin, etoposide, oxaliplatin, and irinotecan. Sometimes, procarbazine and vincristine may be added to CCNU (lomustine) as the PCV regimen, which used to be a first line approach before the supremacy of TMZ. A few other chemotherapy approaches warrant a brief mention. Gliadel is a small wafer that contains the chemotherapeutic drug carmustine (BCNU) and a biodegradable polyanhydride copolymer. Up to 8 Gliadel wafers are implanted in the resection cavity, designed to release BCNU slowly over a period of 2‒3 weeks. Clinical trials results have shown that Gliadel prolonged survival in a statistically significant (albeit modest) manner in both newly diagnosed patients and patients with recurrent glioblastoma multiforme when used as adjunctive therapy to surgery and/or radiation therapy – 13,9 months compared to placebo implants – 11,6 months [42]. Nota bene, Gliadel wafers may have terrible side–effects: seizures, brain edema, wound healing problems, intracranial infections, delay in the administration of other drugs. Chronic administration of chloroquine – 150–300 mg dose of chloroquine daily starting 1 day after surgery–greatly enhanced the response of GBM to antineoplastic treatment. Because the cytotoxicity of chloroquine on malignant cells is negligible, these favorable results appear mediated by its strong antimutagenic effect that precludes the appearance of resistant clones during radiotherapy and chemotherapy [43]. Other experimental approaches to the chemotherapy of GBM include: anti–angiogenic agents like anti–VEGF monoclonal antibodies, e.g. Bevacizumab (Avastin), anti–FGF antibodies, monoclonal antibodies targeting EGFR like Erlotinib and Gefitinib; inhibitors of other tyrosine kinase receptors (e.g. PDGFR); inhibitors of kinesin Eg5; mTOR inhibitors like: Everolimus, Sirolimus, Temsirolimus; farnesyl–transferase inhibitors like: Tipifarnib, Lonafarnib. A promising avenue of individualized research is an antitumoral vaccine made from specific proteins isolated from glioblastoma cells following resection, in order to generate a specific immune response to that particular tumor [44].

## Authors' experience

We analyzed data from 118 patients with a final histopathological diagnosis of GBM, treated in the Neurosurgery Clinic of the University Hospital, Bucharest, in the last 6 years (2003–2008). Patients received either surgery or biopsy and adjuvant therapy in the form of radiotherapy, chemotherapy, following standard protocols. Overall, the median survival was 49 weeks. There was a significant difference in survival between the 73 patients who have undergone resection (57 weeks) and the 45 patients who were only biopsied (38 weeks) (p<0.01). Obviously, this difference is at least partly due to selection bias, as patients with multifocal and large bilateral tumors (‘butterfly GBM’) who presented in an advanced consumptive state (poor Karnofsky scores) or who were not good candidates for extensive open surgery due to advanced age and comorbid disorders, were more often offered biopsy. Table 1 presents prognostic factors and survival in our cohort as a function of age, confirming that, contrary to many other malignancies, younger is better for GBM patients.

## Management of recurrent GBM

Semantically [3], there are two distinct clinical entities:

1. **Tumor *recurrence***: after complete surgical removal of the visible mass, documented radiologically (MRI), the tumor re–appears after a variable free interval, usually within 2 cm of the original site (correlate with [23]), although 10% of patients may develop new lesions at distant sites.
2. **Tumor *progression***: after a subtotal excision, the mass never disappears completely from imaging studies and after a while there is radiological documentation of an increase in tumor size.

There are many factors favoring recurrence: subtotal excision (either erroneously or intentional, in order to protect eloquent areas); tumor multicentricity; histopathological features of aggressiveness (extensive angiogenesis, high mitotic index etc.), genetic features (deregulated genome with many mutations accumulated); clinical factors (e.g. Karnofsky score, age, comorbidities, other factors that may influence administration of adjuvant therapy) and perhaps most important, the inherent infiltrative nature of GBM–at the time of diagnosis, there are already malignant cells disseminated at a distance from the bulk of the tumor, mocking the scalpel. Recurrence usually means the tumor is becoming more aggressive, genetically and clinically and it has acquired resistance to the adjuvant therapy. For the patients and their families, it is important to do something to offer a longer life expectancy, maintaining at the same time a good quality of life for a reasonable period, without supplementary neurological deficits. For the society, the most embarrassing problem is the costs of different procedures: surgery, chemotherapy, radiotherapy. For the neurosurgical community, reoperation for recurrent malignant gliomas is still a difficult dilemma. So what is to be done for these patients? Unfortunately, additional radiation has limited control on further tumor growth and would potentiate neurologic toxicity. It has been shown that prior chemotherapy had significant correlations with time to tumor progression and survival, but further cycles of chemotherapy are problematic in these patients, due to their biological status and the heightened risk of cumulative myelotoxicity (as the recurrence is usually fast, contrary to some other cancers). Radiosurgery may be an option, for tumors of appropriate size, pattern and location. Open surgery is also to be considered, it could be life saving to patients with lesions that produce increased intracranial pressure and mass effect. From a life quality standpoint, to selected patients with recurrent GBM, repeat surgery could be beneficial and a reasonable therapeutic alternative. If the tumor is better circumscribed on recurrence, extensive removal is indicated, as radical as first-time surgery. On the contrary, patients over 60 years old, with bad preoperative function, low performance status and a short symptom free period after the first surgery, have a bad prognosis. Significant peritumoral edema, symptomatic mass effect, location near eloquent areas are other negative prognostic factors for surgery. Timing is also important. If tumor recurrence occurred within 6 months after a radical resection and radiotherapy, progression or recurrence after second surgery are much quicker; in case a year or more has passed after the initial presentation, it would be worthwhile to excise the recurrent tumor.

## Conclusions

The prognosis of GBM is still dismal. Without treatment, the median survival from the diagnosis is 3 months (death is usually due to cerebral edema and increased intracranial pressure). With maximal treatment, median survival is only 14 months, despite major improvements in neuroimaging, neurosurgery, radiation treatment techniques and the advent of temozolomide– ‘bullets but no magic’– [45]. The survival at 2 years is 16% and at 3'years is 5%. Only approximately one in 5000 GBM patients survives for decades. That being said, patients with GBM are not universally incurable, with an ever increasing albeit small fraction of patients who fight and survive the disease. Progress in improved outcomes for GBM patients will require the identification and development of therapeutics with high specificity for brain tumor cells and that have the ability to access the entire intracranial compartment.

**Table 1:** Table 1

**Age group**	**Median survival (number of patients)**
20–40	68 weeks (19)
40–60	57 weeks (35)
> 60	40 weeks (68)
**Favorable prognostic factors**	**Unfavorable prognostic factors**
Age 20–40	Age > 60
Karnofsky score > 70	Karnofsky score < 70
Resection	Biopsy Only
Single mass	Multifocal tumor
